# Determining the acceptability of a novel One Health vaccine for Rift Valley Fever prior to phase II/III clinical trials in Uganda

**DOI:** 10.1016/j.onehlt.2022.100470

**Published:** 2022-12-06

**Authors:** Alexander Bowmer, Joseph Ssembatya, Mark Okot, Richard Bagyenyi, Stephen Venny Rubanga, Gladys Kalema-Zikusoka

**Affiliations:** London School of Hygiene and Tropical Medicine | Conservation Through Public Health | Uganda Virus Research Institute, Uganda

**Keywords:** Vaccines, Anthropology, RVF, One Health

## Abstract

Several vaccine candidates for Rift Valley Fever (RVF) are in development for use in humans. A promising candidate, ChAdOx1 RVF vaccine, has been developed for use in both humans and animals, and has undergone field trials in livestock in Kenya. We conducted a qualitative study to explore the acceptability of this novel One Health vaccine for Rift Valley Fever prior to phase II/III trials, in two rural Ugandan cohorts between January to June 2020. Data was obtained from 96 semi-structured interviews at Bwindi Impenetrable National Park (BINP) and Kyamulibwa, Kalungu District, in Southern Uganda. The study found that 42% of those interviewed were willing to receive a vaccine that was the same for both humans and animals. 45% of those interviewed said that they would not be willing to receive a One Health vaccine and a further 13% were unsure whether or not they would be happy to receive such a vaccine. Semi-structured interviews were conducted to explore their reasons for and against the acceptability of a novel One Health vaccine to highlight potential barriers to deployment once a vaccine candidate for RVF becomes available.

## 1 Introduction

Rift Valley Fever (RVF) is a neglected re-emerging zoonotic viral infection associated with up to 90% mortality in livestock and 30% mortality in humans. [1,2] It is spread by several species of mosquito found across sub-Saharan Africa and the Arabian Peninsula. RVF outbreaks are infrequent but when they occur, can be unpredictable in nature causing overwhelming human and cattle morbidity and mortality. The most recent outbreaks have been reported in Mayotte (2019), Sudan (2019, 2007), Uganda (2018), Niger (2016), Mauritania (2012), South Africa (2010), Madagascar (2008, 2009), Kenya, Somalia, and Tanzania (2006), Egypt (2003), and Saudi Arabia and Yemen (2000). [3]

Livestock trade, human migration and changing environmental conditions have contributed to the additional spread of RVF. [4] This is because humans and livestock coexist in a strongly interdependent relationship and share or depend on each other for security, shelter, food, water, transport, and related by-products. [5] This interdependence predisposes humans to infection through mosquito bites and contact with, or consumption of, animal products. [6] However, the magnitude of zoonotic transmission cannot be clearly defined due to very low and unreliable human and livestock surveillance for the disease, and poor collective knowledge on how farmers recognise and manage the acute symptoms of RVF. [7,8] This is concerning, as early detection of RVF is crucial to containing an outbreak, when preventative technologies such as vaccines have not yet provided an adequate solution. [9]

Although RVF vaccines for livestock have existed for decades, [[10], [11], [12], [13], [14]] there has been less progress in developing one for humans. Even those developed for livestock have had little success or efficacy. [13] ChAdOx1 RVF, a novel One Health vaccine, is the first RVF vaccine in co-development for human and livestock use and has undergone field trials in livestock in Kenya. [15,16] As Capps and Lederman discuss [17], to understand the role of One Health vaccines, we have to understand the connections between ‘vectors and victims’, a term they use to describe those who are afflicted by a communicable disease and those that transmit them, our shared ecologies and options for control. Zoonoses (diseases transmissible between animals and humans) account for approximately 60% of all infectious pathogens of human beings and 70% of all naturally emerging infectious diseases. [[18], [19], [20]] Underlying factors such as expanding livestock and wild animal trade, human migration and changing environmental conditions have contributed to the emergence and additional spread of zoonotic diseases such as RVF, Ebola Virus Disease, Hendra Virus, Middle Eastern Respiratory Syndrome and Brucellosis, across LMIC contexts. As with humans, advantages to animals affected by diseases such as RVF or Ebola, which have infected gorillas, bush pigs, porcupines and cattle, is protection from the disease and prevention of cross infection. [21] This is known as a ‘shared benefit’ approach. As Towner et al. notes [22], by protecting the health of one species from an infectious pathogen, you in turn benefit another species by preventing the further transmission of the said pathogen. In the case of RVF, due to the risks posed to both human and animal populations, a One Health vaccine approach allows for simultaneous preventative control strategies that prevents both spillover and ‘reverse spillover’ events.

Before progressing to phase II/III trials, it is essential to evaluate community perceptions of using a vaccine that is the same for both humans and animals, especially amongst the study population. Doing so provides opportunity to identify potential barriers to deployment and the time necessary to respond accordingly, using community perspectives as a guide to increase vaccine uptake and co-develop further clinical trials. [23,24]

## 2 Material and methods

We conducted a qualitative study to explore the acceptability of a novel One Health vaccine for Rift Valley Fever prior to phase II/III trials in two rural Ugandan cohorts between January to June 2020. Data was obtained from 96 semi-structured interviews in Bwindi Impenetrable National Park (BINP) and Kyamulibwa, Kalungu District, in Southern Uganda. Selection of participants was conducted using census data and from lists of subsistence livestock farmers provided by community mobilisers. 47 farmers around the BINP Northern sector were selected from four locations in villages where farmers were interviewed and some of their animals sampled, and treated where necessary. Group l was selected in Nkwenda, Buhoma and Mukono. Group 2 in Kyumbugushu, Rubona and lraaro; Group 3 Hamayanja, Byumba and Kazaai; and Group 4 in Nyamishamba and Muchorero. 49 farmers were selected in Kyamulibwa, Kalungu District, through the MRC/LSHTM/UVRI General Population Cohort (GPC). A list of participants was provided by the data manager and community mobilisers, and farmers were selected using snowball sampling. In total, 96 individuals were interviewed, representative of 1421 livestock animals.

First drafts of the interviews were based on a literature review and discussions with health and veterinary health experts at the Uganda Virus Research Institute (UVRI) and NGO Conservation Through Public Health (CTPH). The drafts were pilot tested in the field by research assistants (RA) from UVRI and CTPH and further refined in a participatory process involving the RA's and representatives of the community, namely community health workers and veterinary technicians operating in both areas. Each interview took approximately 45 min and was facilitated by RA's. In Bwindi, RA's were also accompanied by veterinarians and veterinary technicians who took samples from livestock, provided healthcare advice and administered treatment when required. All interviews were recorded on the properties of the interviewees.

## 3 Data analysis

Interviews were audio recorded, transcribed and translated from Bantu (Luganda and Rukiga) into English. Data analysis was completed manually and analysed using Microsoft Word**.** Analysis of transcripts was conducted through an iterative and deductive process, whereby categories regarding attitudes towards a One Health vaccine became apparent.

## 4 Ethical considerations

Ethics approvals were obtained from the Uganda National Council for Science and Technology (UNCST), Uganda Virus Research Institute (UVRI) and the London School of Hygiene and Tropical Medicine (LSHTM). All study participants received an informed consent document and agreed to have their answers anonymised and quoted in publication. All participants received reimbursement for their time. The amount given to participants was decided by community leaders and was in line with the ethical approaches outlined by the UVRI and CTPH. Findings are presented as quotes using the following notation ID. KY or ID. BW. and refer to interview participants with an anonymised identification number.

## 5 Results

The findings of this study provide insight into barriers to vaccine deployment, the acceptability of a novel One Health vaccine, and demonstrate how social science research can be used to inform communication strategies before and during the development of future clinical trials. The study found that 42% of those interviewed were willing to receive a vaccine that was the same for humans, as it is for animals. 45% of those interviewed said that they would not be willing to receive a One Health vaccine and a further 13% were unsure whether or not they would be happy to receive such a vaccine. Reasons for the given answers have been analysed and divided into themes relating to *fear*, *uncertainty*, *experiences of use*, and *necessity*.

### 5.1 Fear

Throughout the interviews we asked farmers how they would feel about receiving a vaccine that was the same for both humans and animals. A prominent theme that emerged was *fear*. Some farmers discussed their fears regarding the novelty of such a vaccine and the need for clear licensing, labelling and safety information if the vaccine administered to both humans and animals was identical in its contents. One farmer stated…

*I would participate if many other people have participated too but with fear, but for you the health workers, it seems you have some ideas about it but for us, we are afraid… because we don't know whether the one meant for the people. We are afraid, because why we are saying so, on the bottle or tin of the animal drugs they indicate the cows, the goats the pigs and the other animals I don't know, why don't they put the picture of the person there?.* ID. KY 17032034.

A number of livestock farmers in Bwindi expressed more specific concerns regarding their fears of receiving such a vaccine, with one farmer stating…

*It will result into death of humans when they are given animal vaccines…* ID. BW. 001.

Similar thoughts were also shared by livestock farmers in the same area who discussed their fears regarding the strength of a vaccine, the intention of such a vaccine, and expressed concerns that they were being treated as animals themselves. Examples included…

*That drug would kill me. If you use an animal drug in a human it is like witchcraft*… ID. BW. 007.

*If someone does that to me and I get to know, I am sure the intention was to kill me… I don't think human drugs can work in animals and vice versa.* ID. BW. 0013.

*I have never heard of that, I would be scared because they say after injecting the goat or cow and it dies people are not even supposed to eat it. If you cannot eat the dead animal after treatment then would give the same drug to humans.* ID. BW. 0012.

*I cannot do that, now I am an animal? Unless they want to kill me. You know the goats and cows have higher volumes of blood than humans because they eat all plants which are medicinal.* ID. BW. 0040.

It became clear during our conversations that *fear* played a central role in the acceptance of a One Health vaccine. Fears of the intentions of those creating it, it being a new technology, and the belief that animal vaccines had to be stronger than that of humans, provoked resistance towards accepting such a product. Our analysis also highlighted very specific fears as highlighted above, which have also been seen in similar studies on vaccine hesitancy more broadly. [[25], [26], [27]]

### 5.2 Uncertainty

Another prominent theme that emerged during analysis was *uncertainty.* Uncertainty was described as a hesitation towards the novelty of a vaccine that could be administered to both humans and animals. One farmer stated…

*It cannot be the same; it has to be prepared differently, because you cannot inject me with that. I know of one thing, there is a spray for wounds which can be used on animals as well as the people, but it's wrong to be used by people on their wounds, but because its effective and you cannot compare the animals to the people, so in my case I do not agree to use it on me! No, no, no…* ID. KY. 17,199,012.

Uncertainty also derived from hesitancies within the community, such as those described by a number of participants regarding their experiences with a HIV vaccine trial conducted in the area…

*I would not be certain of the vaccine, for example, there was a time I heard of an exercise when they were trying to vaccinate people with a trial vaccine for HIV, and I asked myself, HIV is sexually transmitted, I don't know whether you know of other methods of transmission but that is the most common, and after the person had been vaccinated against that, then how will he know that when I did this, I didn't contract the infection and the person I was engaged with was HIV positive, so when it comes to this vaccine, I ask myself that if I'm vaccinated, like say against brucella, in fact, will it work! And if I get problems!.* ID. KY. 17,135,085.

Another farmer furthered this by discussing their uncertainties regarding how testing on both humans and animals can be comparable, a sentiment shared by many in this dataset. For example…

*How can I prove that it is both useful for animals and human beings? I don't think I can believe it, I deny it because treatment and drugs for animals are not the drugs for human beings. There must be a difference. Veterinary is not matching with human drugs.* ID. BW. 0011.

### 5.3 Experiences of use

Previous experiences both positively and negatively affected attitudes towards the introduction of a novel One Health vaccine. An example of this can be seen in one farmers account who stated…

*I don't have any objection to that because, let me give an example of the children, when they are vaccinated and every infection that they vaccinate them against, the children suffer from measles, but the child who is vaccinated is not affected by it, therefore when they come up with the vaccine that is meant to prevent such infections from being transmitted to the people, I don't have any problem with it…. -* ID. KY 01107021.

Experiences of use also generated alternative discussions regarding personal experiences with previously deployed vaccines and community perceptions, as discussed in the following two accounts…

*To take you back a bit, when we were told to vaccinate our children, and we vaccinated them but later after that they started suffering from very many infections, and many people said that the vaccines that were used were not up to the required standards, and I for one, my children experienced the same thing because I was admitted to the health centre several times, so because we are sceptical of the vaccines that are brought now, we don't know how it is transported and what they have put in them…* ID. KY 07466070.

*Some of the people who had cattle didn't take them there because they had their own reasons, like when we have the campaigns to vaccinate our children, some would say that they would not take theirs for vaccination because they don't know why they are vaccinating them again, that they want to infect them.* ID - KY 11100103.

### 5.4 Necessity

A further theme that emerged during analysis was *necessity*. Necessity was discussed as part of the decision-making processes farmers felt they had to make on behalf of those dependant on them for care. In reference to a novel One Health vaccine, farmers questioned the need for, and benefits of, having a single vaccine and not separate for humans and animals…

*If that kind of vaccine comes, it would be good, because if you have bought it or when it is given to you, and you vaccinate the animals as well as vaccinating your people and yourself, because you may vaccinate the animals whereas the infectious germs have remained somewhere near you, and some may be spread through the air, and infect you. So if you vaccinate yourself that means the infection may not affect you as a person and the animal too may not get infected, it could be a good vaccine, because there is the bird flu, if it can infect a person and kill him, then when you vaccinate yourself against those infections, you are sure that you are safe.* ID. KY. 12,952,021.

Concerns were also shared amongst those interviewed with many farmers suggesting that human and veterinary medicines must remain separate. Examples included…

*I think animals have their own vaccine and people also have their own vaccine*. I.D. BW 004.

*I feel bad and if I see that it is for animals, I tell you right away not to give me the animal vaccine. I would refuse, for animals is different from that of humans.* I.D. BW 009.

## 6 Discussion

The far-from-universal willingness to accept a novel One Health vaccine for RVF in our two data sets is cause for concern. Farmers that were more willing to accept the vaccine typically had increased contact with both human and animal healthcare workers, and greater access to vaccines that were supplied by veterinary technicians, community health workers or sold in local drug stores. Where farmers had previously attended workshops on animal husbandry and were educated on the risks posed to their health without proper consideration for the hygiene of their animals, there was increased acceptance of ChAdOx1 RVF. This is likely because these farmers possess a greater awareness of disease transmission pathways and saw value in a vaccine that could prevent the onset of disease in both their animals and themselves.

Those who expressed a complete unwillingness to accept such a vaccine were typically farming in remote areas surrounding Bwindi Impenetrable Forest. Awareness of vaccination as a form of providing prophylactic and therapeutic care for human and animal health was reported by all those interviewed and visual stimulus of a vaccine was provided as a prompt to ensure clarity and to prevent confusion regarding what a vaccine was. A distrust in the role of vaccines was most commonly reported. Farmers reported concerns that a vaccine that was the same for both themselves and their animals would harm their health, make themselves, their children and/or animals sterile, and contradicted previous advice they had been given with regards to not mixing human and animal medication. Fear that a vaccine such as ChAdOx1 RVF would be too strong for human use was also a prominent concern reported. Due to the size of their animals, farmers often expressed concern that the vaccine would harm them because their bodies would not be able to cope and as a result, it may kill them. Farmers also questioned the necessity for such a vaccine, as many believed that they had not experienced RVF, would sell on their sick animals to market instead of calling out a technician or purchasing their own vaccines, or they believed a good diet and animal husbandry was all that was needed to prevent the onset of disease.

Our findings suggest that prior to phase II/III clinical trials for ChAdOx1 RVF, or any novel One Health vaccine in development, we must acknowledge community concerns and move beyond singular narratives that vaccines are safe and effective to address the complex web of factors that influence the acceptability and uptake of new vaccines. [[28], [29], [30]] As Heidi et al. suggest, strategies to increase the acceptability of vaccines should directly address community specific concerns, misinformation, rumours and misconceptions. [23,24] Researchers have demonstrated promising interventions and community engagement strategies in different contexts, but these strategies have not been applied to human/animal vaccines and further research is required in this area.

As Larson et al. further note, trust is an intrinsic and potentially modifiable component of successful uptake of vaccines. Our findings show that trust in community healthcare providers, both those attending to human and animal health, is strongly associated with increased acceptability of a novel One Health vaccine. [31,32] However, it is important to consider that reporting one's willingness to accept a novel One Health vaccine may not necessarily be a good predictor of acceptance or trust in vaccines, as vaccine decisions are multifaceted and can change over time. [33] Similarly, although our data set included 96 ruminant livestock farmers, this study would benefit from a larger sample size in other locations across the ‘cattle corridor’ between Masaka and Kampala, where further trials of ChAdOx1 RVF are likely to take place. Given staff time restraints, double coding of all interviews was not possible. This is a further acknowledged limitation of this study.

## 7 Conclusion

Addressing the hesitancies towards a novel One Health vaccine presented in our interviews requires more than building trust. It requires targeted and community specific engagement to identify, understand and address barriers to vaccine deployment. Community engagement should therefore be conducted prior to, during and after Phase II/III trials commence to increase acceptability of ChAdOx1 RVF. As our data has shown, local healthcare workers should be at the forefront of engagement as they are trusted providers of healthcare advice and services in these communities. Operational recommendations should also be made prior to trials. This includes the packaging and licensing of the vaccine, and the information provided to participants prior to recruitment which should clearly address why the vaccine can be administered to both humans and animals. In the context of emerging RVF outbreaks, the findings of this study help us to identify barriers to vaccine deployment and optimise uptake of a novel One Health vaccine candidate, such as ChAdOx1 RVF.

## Declaration of Competing Interest

The authors declare that there is no conflict of interest.

## Data Availability

The data that has been used is confidential.
